# How do hospital administrators perceive cardiac rehabilitation in a publicly-funded health care system?

**DOI:** 10.1186/1472-6963-13-120

**Published:** 2013-03-28

**Authors:** Sherry L Grace, Sabrina Scarcello, Janet Newton, Blair O’Neill, Kori Kingsbury, Tiziana Rivera, Caroline Chessex

**Affiliations:** 1 Department of Kinesiology and Health Science, Bethune 368, York University, 4700 Keele St, Toronto, ON, M3J 1P3, Canada; 2 Peter Munk Cardiac Center Cardiovascular Rehabilitation & Prevention Program, Toronto General Hospital, University Health Network, 200 Elizabeth St, Toronto, ON, M5G 2C4, Canada; 3 Division of Cardiology, Mazankowski Alberta Heart Institute, University of Alberta, 8440 112 St. NW, Edmonton, AB, T6G 2B7, Canada; 4 Cardiac Care Network of Ontario, 4100 Yonge St., Suite 502, Toronto, ON, M2P 2B5, Canada; 5 MacKenzie Health, 10 Trench St, Richmond Hill, ON, L4C 4Z3, Canada; 6 Faculty of Medicine, University of Toronto, 1 King’s College Circle, Toronto, ON, M5S 1A8, Canada

**Keywords:** Hospital administrators, Attitude of health personnel, Outpatient health services, Rehabilitation centers, Cardiac care facilities

## Abstract

**Background:**

Patient and provider-related factors affecting access to cardiac rehabilitation (CR) have been extensively studied, but health-system administration factors have not. The objectives of this study were to investigate hospital administrators’ (HA) awareness and knowledge of cardiac rehabilitation (CR), perceptions regarding resources for and benefit of CR, and attitudes toward and implementation of inpatient transition planning for outpatient CR.

**Methods:**

A cross-sectional and observational design was used. A survey was administered to 679 HAs through Canadian and Ontario databases. A descriptive examination was performed, and differences in HAs’ perceptions by role, institution type and presence of within-institution CR were compared using t-tests.

**Results:**

195 (28.7%) Canadian HAs completed the survey. Respondents reported good knowledge of what CR entails (mean=3.42±1.15/5). Awareness of the closest site was lower among HAs working in community versus academic institutions (3.88±1.24 vs. 4.34±0.90/5 respectively; p=.01). HAs in non-executive roles (4.77±0.46/5) perceived greater CR importance for patients’ care than executives (4.52±0.57; p=.001). HAs perceived CR programs should be situated in both hospitals and community settings (n=134, 71.7%).

**Conclusions:**

HAs value CR as part of patients’ care, and are supportive of greater CR provision. Those working in community settings and executives may not be as aware of, or less-likely to value, CR services. CR leaders from academic institutions might consider liaising with community hospitals to raise awareness of CR benefits, and advocate for it with the executives in their home institutions.

## Background

Cardiovascular disease (CVD) is the leading cause of morbidity and mortality globally. In 2008, 30% of deaths worldwide were due to CVD [1]. With improvements in treatment, there are more people living chronically with CVDs, such that CVD represents 10% of the global burden of disease [1]. Moreover, CVD is one of the most expensive health conditions worldwide. CVD is the leading economic cost in Canada with total direct (i.e., hospital care, drugs, physician care, other institutional care) and indirect costs (i.e., mortality, and short and long-term disability) amounting to CAD$20.9 billion in 2005 [2]. In 2003, CVD was estimated to cost the European Union Euro€169 billion [3]; in 2008, the American Heart Association estimated that the total cost of CVD was US$297.7 billion [4]. Furthermore, between 2012 and 2030, total direct medical costs of CVD are estimated to triple in the US, from $309 billion to $834 billion.

Cardiac rehabilitation (CR) is a cost-effective outpatient chronic disease management program that can successfully address this chronic CVD burden [5]. CR participants undergo comprehensive medical assessment to optimize risk reduction, receive an individually-tailored exercise prescription, participate in supervised exercise, receive education, and behaviour change counseling [6]. It is well-established that CR participation is related to lower overall and cardiovascular mortality over several years when compared to usual care [7]. The clinical benefits of CR are well-documented and include improvements in exercise capacity, risk factors, health behaviours, and quality of life [8]. Participation in CR also has beneficial effects for the healthcare system, by decreasing the burden of rehospitalizations and revascularization procedures [9].

Based on this evidence, clinical practice guidelines recommend referral to CR [10]. Given the importance of early, universal access and continuity of care, it has been recommended that patients be systematically referred to CR before hospital discharge [11]. Despite these recommendations however, CR is infrequently used. Approximately only 15-30% of eligible patients access CR [12].

Patient- [13-18] and provider- [16-18] related factors affecting access to CR have been extensively studied. System-level factors have been more recently investigated [19], and factors identified included level of integration of CR within the hospital setting, degree of automation of CR referral in the inpatient setting, and capacity constraints. However, to the best of our knowledge, there have been no studies that have examined health-system administration factors specifically, in relation to CR utilization. These are particularly important, considering CR programs are generally located within hospitals, or at local community centres but with hospital affiliation. Hospital administrators (HAs) plan, organize, direct, and coordinate health services, including inpatient care and ambulatory clinics [20]. Moreover, HAs set budgets for healthcare services. Clearly, staffing, space, availability of equipment, operating hours and financing are all key to CR provision. However, little is known about HAs’ knowledge, perceptions and endorsement of CR programs within hospitals. Thus, the objectives of this study were to investigate HAs’ awareness and knowledge of CR, perceptions regarding resources for CR, and attitudes toward and implementation of inpatient transition planning for outpatient CR services. Furthermore, subgroup analyses were performed to examine differences in HAs’ perceptions of and attitudes toward CR as a function of institution type, HA role and presence of within-institution CR.

## Methods

### Design and procedures

The study was observational and cross-sectional in design. Ethics approval was obtained from the institutional research ethics board at York University. Identification of HAs was facilitated through the Ontario Hospital Association (OHA), Cardiac Care Network of Ontario (CCN) and Canadian Healthcare Association (CHA) databases. CR service pay models vary by province in Canada, with some provinces supporting CR (e.g., Ontario) and others not (e.g., Quebec). Participants were recruited via email and surface mail. Recruitment letters and emails were delivered between October 2011 and November 2011. Voluntary completion of the survey constituted informed consent. All participants were assigned a survey identification number to ensure anonymity.

Members of the OHA and CHA were mailed a letter inviting them to participate in the study. Members completed the paper-and-pencil survey or accessed an online version of the survey through a link to Hosted Survey (http://www.hostedsurvey.com/home.html) that was provided in the letter. Letters were again mailed two weeks after the initial mailing as a reminder to complete the survey. Members of the CCN HA committee were sent an email to introduce the study and solicit their participation. They were able to access the survey directly through a link to Hosted Survey that was provided in the email. Similarly, all members of the CCN committee were re-emailed 2 weeks after initial contact as a reminder to complete the survey.

### Participants

Canadian HAs working in an area related to cardiovascular medicine were eligible to participate in the study. There were no exclusion criteria for the present study. Six hundred and seventy-nine surveys were sent: 531 (78.2%) HAs from the CHA and 128 (18.9%) HAs from the OHA were mailed hard copy surveys, and 20 (2.9%) members of the CCN HA committee were emailed.

### Measures

The survey was developed based on a review of the literature, and with input with HAs on the investigative team. It consisted of investigator-generated items with open and closed-ended response options, as well as Likert-type response options. In the first part of the survey respondents self-reported demographic information, specifically their job title/position (e.g., executive, director, manager from a list of forced-choice response options), highest degree obtained, year they graduated from their most advanced degree, and sex. Respondents were also asked to report the type of institution in which they worked from a list of forced-choice response options (e.g., community hospital, academic centre). They also responded to questions on CR programming specific to their institution. In the second part of the survey, respondents rated their knowledge, awareness and perceptions of CR.

### Statistical analyses

Statistical analyses were conducted using IBM Statistical Package for the Social Sciences version 19.0. A descriptive analysis of all variables was performed. Student’s t-tests were computed to assess relationships between: [1] respondents’ knowledge and awareness of CR in relation to whether they were employed at a community or non-community institution; [2] respondents’ perceptions of the importance of CR and clinical benefits in relation to whether they were in an executive or non-executive role, and [3] respondents’ attitudes toward CR in relation to whether or not the institution where they worked had a CR program. A value of p<.05 was used to denote significance.

## Results

### Respondent characteristics

The sample included 195 respondents, of which 124 (63.6%) completed the survey on paper. Of the mailed surveys, 15 (2.3%) were returned because the address was not valid. Of the online surveys, 2 (0.1%) email addresses were invalid and no further contact information was available. Two (0.1%) email contacts were no longer working in cardiac HA and were removed from the list. The overall response rate was 28.7% (i.e., 195/679).

Respondent characteristics are summarized in Table 1. Other types of institutions indicated by respondents included integrated health care agencies (n=1), regional health authorities (n=1), regional cardiac programs (n =1), and family health teams (n=1). Seventeen of 22 respondents reporting an “other” job title or position did not specify, but other responses included “health service administrator” (n=2, 9%), and infection prevention and control nurse (n=1; 4.5%). Overall, 94 (51.4%) HAs worked in an institution with a CR program.

**Table 1:** **Respondent characteristics, *N*=195**

**Characteristic**	**n (%) /**
	**mean ± SD**
Sex, female (%)	122 (64.6)
Province of work, in alphabetical order	
Alberta (%)	14 (7.2)
British Columbia (%)	6 (3.1)
Manitoba (%)	10 (5.2)
New Brunswick (%)	4 (2.1)
Newfoundland and Labrador (%)	2 (1.0)
Nova Scotia (%)	1 (0.5)
Ontario (%)	139 (71.6)
Prince Edward Island (%)	1 (0.5)
Quebec (%)	13 (6.7)
Saskatchewan (%)	2 (1.0)
Northwest territories (%)	2 (1.0)
Institution type	
Community hospital (%)	128 (66.3)
Academic health sciences centre (%)	58 (30.1)
Quaternary/Tertiary facility / Cardiac Centre (%)	25 (13.0)
Rehab/outpatient institution (%)	7 (3.6)
Government (%)	1 (0.5)
Other (%)	5 (2.6)
Job title / position	
Executive leadership (%)	84 (43.3)
Manager (%)	33 (17.0)
Medical director (%)	23 (11.9)
Clinical director (%)	17 (8.8)
Supervisor/coordinator/ team lead (%)	15 (7.7)
Other (%)	22 (11.3)
Years of service in current role	
<1 year (%)	20 (10.4)
1-5 Years (%)	77 (39.9)
6-10 years (%)	41 (21.2)
>10 years (%)	55 (28.5)
Primary clinical area	
Cardiac (%)	66 (34.4)
Non-clinical focus (%)	54 (28.1)
Non-cardiac-specific area	72 (37.5)*
General (%)	25 (48.1)
Multiple areas (%)	6 (11.5)
Acute care (%)	5 (9.6)
Complex continuing / long-term care (%)	5 (9.6)
Emergency (%)	4 (7.7)
Surgery (%)	3 (5.8)
Other (%)	4 (7.7)
Highest level of education	
Graduate degree (%)	74 (38.5)
Medical school (%)	50 (26.0)
Undergraduate degree (%)	38 (19.8)
College diploma (%)	28 (14.6)
High school (%)	2 (1.0)
Graduation year (mean ± SD)	1991.4 ±12.0

*n=20 responses missing or not applicable.

SD=standard deviation.

### HAs’ Attitudes and perceptions of CR

Table 2 summarizes respondents’ knowledge and awareness related to CR. On average respondents reported that they had quite good knowledge of what CR entails, and knowledge of the location of the closest CR program was ‘very good’. Respondents employed at a non-community-based institution (e.g., Academic Health Sciences Centre) reported significantly greater awareness with respect to the location of the closest CR program and the use of CR by patients in their institution. Respondents who worked in non-community settings were also significantly more likely to perceive that their colleagues had a good level of CR awareness.

**Table 2:** Hospital administrators’ mean CR knowledge and awareness (±standard deviation), by type of institution where employed

**Item**	**Community hospital (n=128; 65.6%)**	**Non-community (n=67; 34.4%)**	**Total N=195**
1. My awareness of the location of the closest rehab program to the institution where I am employed	3.88 ±1.24	4.34±0.90	4.03±1.16††
2. My knowledge of what CR entails	3.34±1.20	3.60±1.03	3.42±1.15
3. My awareness of the use of CR by patients in the institution where I am employed	3.01±1.31	3.44±1.18	3.15±1.28†
4. My colleagues’ level of CR awareness	2.94±1.12	3.27±0.87	3.05±1.06†

Note: Items were scored on a scale from 1 ‘poor’ to 5 ‘excellent’.

††p=.01, †p<.05.

Table 3 summarizes respondents’ perceptions and ratings of their institution’s perspective of CR with regard to clinical benefit. Respondents reported that their institutions perceived CR to be important for patients’ outpatient care, and that access to CR plays an important role in reducing patient length of stay. Respondents in non-executive roles (e.g., Manager, Clinical Director) perceived significantly greater importance of CR for patients’ outpatient care, and that CR programs were important for reducing patient re-admissions than executive HAs. Finally, respondents in executive positions perceived significantly less value in serving patients with other vascular conditions within CR programs than non-executive HAs.

**Table 3:** Hospital administrators’ mean perceptions of CR (± standard deviation), by job title or position

**Item**	**Executive (n=84, 43.1%)**	**Non-executive (n=111, 56.9%)**	**Total N=195**
1. Your perceptions of the importance of CR for patients’ outpatient care	4.52±0.57	4.77±0.46	4.66±0.53††
2. Your perceptions of the role of CR programs in reducing patient re-admissions	4.34±0.59	4.57±0.53	4.47±0.57†
3. Your institution’s perceptions of the importance of CR for patients’ outpatient care	4.22±0.68	4.15±0.80	4.18±0.73
4. Your perceptions of the value of serving patients with other vascular conditions within CR programs	3.94±0.85	4.26±0.71	4.12±0.79†
5. Your perceptions of the role of CR access in reducing patient length of stay	3.95±0.84	4.14±0.84	4.06±0.85

Note: Items were scored on a scale from 1 ‘Not even considered’ to 5 ‘Extremely Important’.

††p=.001, †p<.01.

Table 4 summarizes respondents’ attitudes regarding CR. HAs strongly agreed that CR programs provide benefits beyond what primary care can offer, and were concordant that their local CR program was of good quality, and promoted sustained behavioral changes that improved patient health outcomes. They reported that it was the hospital’s responsibility to provide inpatients with the information they need to access CR, and that Ministries of Health should provide more funding for CR. On average, HAs disagreed that inpatient staff have more important duties than referring patients to CR. They were not skeptical of the benefits of CR, and perceived that provincial health insurance should cover CR services. Nevertheless, there were some significant differences in attitudes regarding CR among HAs working within institutions that have a CR program versus those who are not. For instance, HAs working in institutions without a CR program were significantly more likely to agree that they did not have enough space for CR, that CR services could be pared back when budget reductions were required. They were also significantly less likely to agree that government should provide more funding for CR programs, or that current levels of government funding for CR will be sustained over time.

**Table 4:** Hospital administrators’ mean attitudes regarding cr (±standard deviation), by presence of cardiac rehabilitation program at the institution where they work

**Item**	**CR program (n=93; 51.4%)**	**No CR (n=88; 48.6%)**	**Total (N=195)**
1. CR programs provide benefits beyond what primary care providers can offer	4.39±0.75	4.77±5.68	4.55±3.88
2. CR programs promote sustained behavioural changes that improve their health outcomes	4.39±0.64	4.23±0.55	4.31±0.60
3. It is the hospitals’ responsibility to provide all eligible inpatients with the information they need to begin an outpatient CR program	4.33±0.91	4.13±0.84	4.22±0.88
4. The closest available CR program is of good quality	4.36±0.82	4.01±0.83	4.20±0.83††
5. The government should provide more funding for CR programs	4.24±0.82	3.85±0.85	4.04±0.84††
6. Ministry funding models are a financial disincentive to CR provision	3.31±1.08	3.37±1.06	3.33±1.05
7. It is likely that government funding for CR programs will be sustained over time	3.36±1.01	3.05±0.88	3.23±0.96†
8. We do not have enough space to run a CR program at my institution	2.36±1.33	3.49±1.28	2.90±1.40†††
9. CR services are generally one of the first programs to be cut-back when we make budget reductions	2.52±1.07	3.20±0.98	2.78±1.08†††
10. Patients and their families should be responsible for their own health behavior changes and risk reduction self-management post-hospitalization	2.43±1.09	2.72±1.09	2.58±1.09
11. Scarce healthcare dollars should not be spent on outpatient care at the expense of acute care	2.06±1.26	2.19±1.01	2.12±1.13
12. Healthcare providers on the cardiac floor have other more important clinical duties than to refer patients to CR	1.71±0.91	1.65±0.73	1.70±0.83
13. Provincial health insurance should not cover CR services for cardiac patients post-hospitalization	1.47±0.75	1.78±0.90	1.61±0.84†
14. I am skeptical about the benefits of CR programs	1.28±0.52	1.65±0.70	1.47±0.65†††

Note: Items were scored on a scale from 1 ‘Strongly Disagree’ to 5 ‘Strongly Agree’.

†††p=.001, ††p=.01, †p<.05.

When asked where CR programs should be situated, HAs most-frequently responded in both hospitals and community settings (n=134, 71.7%), followed by community settings (n=42, 22.5%), and hospitals (n=9, 4.8%). Two responded they should be situated in other settings (n=2, 1.1%), with 1 respondent specifying that CR programs should be situated in regional hospitals.

Participants were provided the average cost of CR in Ontario [21], and asked to report whether they perceived it to be a good use of healthcare dollars. With respect to the $1500CAD cost of CR per patient, 159 (85.5%) respondents perceived this to be a good use of healthcare dollars, 23 (12.4%) respondents perceived this to be a moderately-good use, and 4 (2.2%) respondents did not perceive this to be a good use of healthcare dollars. In terms of an estimated $250,000CAD average annual cost to run a CR program, 153 (83.2%) respondents perceived this to be a good use of healthcare dollars, 25 (13.6%) respondents perceived this to be a moderately-good use of healthcare dollars, and 6 (3.3%) respondents did not perceive this to be a good use of healthcare dollars. When asked to imagine their institutions’ perspective on who should pay for CR (respondents were asked to check all that apply), 107 (57.2%) reported that hospitals’ Ministry of Health-allocated funding envelope should pay for CR services, 107 (57.2%) reported the Ministry of Health Promotion, 44 (23.5%) reported community government organizations, 43 (23.0%) reported the hospitals’ global budget, 37 (19.8%) reported patients, 28 (15.0%) reported community non-profit agencies, and 14 (7.5%) reported a charitable Foundation. Seven (3.7%) reported other funding sources, of which 2 (33.3%) specified a combination of the above, 1 (16.7%) specified the hospital and community, 1 (16.7%) specified community gyms, 1 (16.7%) Local Health Integration Networks (i.e., regional health authority in Ontario), and 1 (16.7%) specified private insurance plans.

When asked for their perspective on an acute care institution’s responsibility for specialized outpatient services, 93 (50.3%) respondents reported that an acute care institution should provide linkages to outpatient services, 86 (46.5%) respondents reported that the full continuum of care should be included in the patient care map, and 6 (3.2%) respondents reported that hospitals should focus on acute care only.

Overall, 94 (50.0%) HAs’ worked in an institution with an inpatient cardiac unit. Of those HAs, 55 (61.1%) rated inpatients’ access to CR in their area as ‘excellent’, 29 (32.2%) as ‘limited’, and 6 (6.7%) as ‘poor’. When asked about CR referral practices, 73 (83.0%) reported there were processes in place at their institution to systematize inpatient referral to CR. Of these responses, 15 (31.9%) described strategies that would not generally be considered “systematic” [22], where a physician independently completes and submits a CR referral form. Of the remaining respondents who specified referral strategies, 25 (53.2%) described an automatic referral approach, 3 (6.4%) indicated that referral was a standard part of a post-discharge follow-up clinic, 2 (4.3%) described patient contact at the bedside prior to hospital discharge, and 2 (4.3%) indicated a combination of automatic referral and patient contact. When asked how often the agenda at meetings in their institution included matters related to inpatient transition to outpatient care, most HAs estimated often (i.e., monthly; n=34, 40.0%), while 31 (36.5%) reported it was sometimes discussed (i.e., semi-annually), and 20 (23.5%) reported it was very infrequently to never discussed. Finally, when asked whether physicians and medical trainees are encouraged to refer inpatients to CR, 60 (68.2%) reported ‘yes, systematically’, 26 (29.5%) reported ‘yes, although this is not actively promoted or facilitated’ and 2 (2.3%) reported ‘no’.

Overall, 93 (51.4%) HAs worked in an institution with a CR program. Of these HAs, when asked whether the programs had dedicated funding, 60 (69.0%) reported ‘yes’, 16 (18.4%) reported ‘somewhat’, and 11 (12.6%) reported ‘no’. When asked to report how they perceived their institutions’ perspective on the scope and size of the CR program from 1 of 3 response options, 52 (59.1%) reported ‘It is under-funded and struggling to provide care to referred patients, but the institution would provide more support if funding was available’, 32 (36.4%) reported ‘It is fully supported and can manage patient volumes’, and 4 (4.5%) reported ‘It is under-funded and struggling to provide care to referred patients, but the institution has no intention to support the program’.

## Discussion

A recent American Heart Association science advisory [23] sets out key recommendations to increase inpatient referral to CR, which includes the importance of liaison with administrative structures in each facility. This study correspondingly investigated HAs’ attitudes and perspectives towards CR, given the role they play in contributing to the under-utilization of CR broadly has been scantly investigated [19]. Overall, HAs endorsed CR as an important component of outpatient care, however CR may not be getting the support it needs from the top boardrooms in Canada.

Results of this study provide insight into some broader questions related to the role of hospitals in CR provision. Most HAs in Canada perceive that CR programs should be situated in hospitals and in community. Indeed, such a model of care would ensure access to supervised and specialized care for high-risk outpatients within academic hospitals, but at the same time ensure broader access for lower-risk patients to home-based and lower-cost CR models in the community where patients could transition to self-management in collaboration with primary care (considering HAs also perceived that CR is needed over and above what can be provided to patients with CVD in primary care). Indeed, a recent presidential advisory from the American Heart Association [24] highlights the importance of alternative CR program models, including interventions delivered over the internet [25]. While the focus of the current survey was on hospital-based programs given this is HAs’ domain of expertise, clearly HAs also recognize the need for alternatives to facility-based services. It is unfortunate however that, despite evidence supporting the efficacy of home-based programs [26], they have not been broadly implemented [27,28]. This may be related to issues of reimbursement for these alternative programs.

With regard to the question of costs and funding, well over three-quarters of HAs perceived the expenses required for running CR to be a valuable use of healthcare dollars. Most HAs perceived that government Ministry’s should be paying for CR, but approximately one-quarter perceived community agencies should be paying, and one-fifth thought that patients should be paying. When compared with the funding reality in Ontario for instance, this is somewhat comparable considering 90% of program funding comes from government sources [21], and patients are generally paying some fees associated with patient education materials or exercise monitoring devices [29].

Only approximately one-third of HAs perceived their within-institution CR programs to have sufficient funding to manage patient volumes. HAs perceived that the government should provide more funding for CR programs, but also perceived it is not likely that even current funding levels will be sustained. The cost-effectiveness of CR has been demonstrated [30,31], and arguably increasing CR capacity would provide a substantive return on investment. Clearly funding models to support CR widely vary. However, HAs are key to ensuing that essential program funds are protected to support CR sustainability. By understanding HAs attitudes and perceptions of CR, we can better advocate for and position CR to complete the patient journey.

Caution is warranted when interpreting these results, due to selection and measurement bias in particular. The response rate was low, suggesting that the perceptions herein may not be representative of those of the larger population of HAs. However, emerging evidence suggests nonresponse bias may be less impactful than previously thought [32]. Second, with regard to measurement, HAs were reporting perceptions only, and in some cases were reporting their perceptions’ of the institutional culture, and these may be biased by expectation or recall. Moreover, self-report of job title or role (i.e., executive vs. director) may not be comparable due to differences in nomenclature between organizations. Third, the design was cross-sectional and therefore no causal conclusions should be drawn. Fourth, multiple comparisons were undertaken, thus increasing the risk of error.

## Conclusions

In conclusion, Canadian HAs value CR as part of patients’ care but funding limitations may restrict the provision of CR services. Overall responses suggest that HAs are supportive of greater CR provision, however those working in community settings and executives who hold major decision-making powers may not be as supportive. It may be warranted for CR leaders from academic institutions to consider liaising with community hospitals in their region to raise awareness of the benefits of CR, and also to advocate for CR with the top executives in their home institutions to make sure they are on the regular meeting agendas.

## Abbreviations

CR: Cardiac rehabilitation; HA: Hospital administrator; CVD: Cardiovascular disease; OHA: Ontario hospital association; CCN: Cardiac care network of Ontario; CHA: Canadian healthcare association.

## Competing interests

The authors declare that they have no competing interests.

## Authors’ contributions

SLG, JN, BO, KK, TR, and CC contributed to the conception and design of the study. SLG and SS participated in analysis and interpretation of the data and drafted the manuscript. All authors read and approved the final manuscript.

## Pre-publication history

The pre-publication history for this paper can be accessed here:

http://www.biomedcentral.com/1472-6963/13/120/prepub
